# *Lactobacillus johnsonii* JERA01 activates macrophages and increases Th-1 T cell population in mouse small intestine

**DOI:** 10.1371/journal.pone.0320946

**Published:** 2025-04-24

**Authors:** So Yeon Ahn, Hong-Gu Joo, Eun-Ju Ko

**Affiliations:** College of Veterinary Medicine and Veterinary Medical Research Institute, Jeju National University, Jeju, Republic of Korea; CNRS UMR 8576 INSERM U1285 University of Lille, FRANCE

## Abstract

*Lactobacillus johnsonii* is a commensal bacterium isolated from the vaginal and gastrointestinal tracts of vertebrate hosts, including humans. It is a potential anti-inflammatory bacterium. As reported in many animal studies, *L. johnsonii* supplementation reduces inflammation in the intestine and enhances the epithelial barrier. However, in this study, we observed immunostimulatory effects of heat-killed *L. johnsonii* JERA01 (LJ) supplementation on antigen-presenting cells, such as dendritic cells and macrophages, in mice. LJ pretreatment increased the expression of maturation markers and TNF-α, IL-6, IL-12p40 production in bone marrow-derived dendritic cells and macrophages (BMDCs and BMDMs). Co-culture of LJ-pretreated BMDCs or BMDMs with lymphocytes enhanced IFN-γ production *in vitro*. Oral LJ (10^8^ CFU) supplementation induced macrophage infiltration into the peritoneal cavity and Peyer’s patch at 12-h after administration, resulting in an increase in the population of IFN-γ-producing T cells in the Peyer’s patch. Our investigation revealed the effects of LJ, which activates macrophages and increases the Th-1 T cell population in the intestine, implying the possibility of using *L. johnsonii* as an immune stimulator.

## Introduction

Probiotics, which are available as supplements or food products, have become a key component of functional foods owing to their potential health benefits [1]. Commonly used strains, primarily from the *Bifidobacterium* and *Lactobacillus* genera, exhibit notable health effects through various mechanisms [2,3]. Probiotics prevent pathogens by improving the intestinal barrier and modulating the mucosal and systemic immune barrier [4]. Species such as *L. reuteri*, *L. plantarum*, *L. gasseri*, and *L. rhamnosus* show antiviral effects by enhancing the antiviral response, which is mainly induced by the activation of antigen presenting cells (APCs) and the subsequent Th-1 response [5–9]. *Lactobacillus* is known to interact with immune cells via toll-like receptors (TLRs), but the mechanism and effects vary among species and strains [10–13]. Therefore, it is essential to understand the specific effects of the different strains to maximize their health benefits.

*Lactobacillus johnsonii* is a common probiotic species [14] and commensal bacterium isolated from the vaginal and gastrointestinal tracts of vertebrate hosts, including humans [15–17]. Some studies have indicated that it possesses potential anti-inflammatory properties and can decrease intestinal inflammation [18,19]. A study by Jia et al. showed that *L. johnsonii* alleviates colitis through IL-10 activation of CD206+ macrophages via the TLR1/2-STAT3 pathway in mice [19]. However, its immunostimulatory effects have rarely been studied. In this study, we evaluated the *in vitro* and *in vivo* effects of heat-killed *L. johnsonii* JERA01 (LJ) on mouse APCs after its oral administration in mice. Although both live and heat-killed *Lactobacillus* spp. have health benefits [2,20–22], we used heat-killed bacteria in this study to avoid safety issues with live bacteria and to provide pharmaceutical advantages in terms of transport and storage [21]. In contrast to the above-mentioned results, the administration of LJ JERA01 resulted in M1 macrophage activation and enhanced the Th-1 T cell population in the intestine, thereby suggesting immunostimulatory effects.

Our findings indicate a variation in the effects of *L. johnsonii* within strains and provide insights into the potential applications of *L. johnsonii* not only in anti-inflammatory treatment but also as an immune stimulator, such as vaccine adjuvants.

## Methods

### Animals and reagents

Seven-to-twelve-week-old female C57BL/6 and Balb/c mice were purchased from SAMTAKO and maintained at Jeju National University Animal Facility. All mouse experiments were performed according to the guidelines of the Jeju National University approved Institutional Animal Care and Use Committees (IACUC) protocol (2020–0050). *L. johnsonii* was provided by SAMDA Co. (Jeju, Republic of Korea) and heat-killed at 90 °C for 2 h in a heating block.

### Bone marrow-derived dendritic cells and macrophage culture

Bone marrow-derived dendritic cells (BMDCs) and bone marrow-derived dendritic macrophages (BMDMs) were generated from the bone marrow cells of Balb/c mice. Bone marrow cells were cultured in Roswell Park Memorial Institute (RPMI) 1640 medium containing 10% fetal bovine serum (FBS) and 1× antibiotic-antimycotic (complete media) supplemented with 20 ng/mL mouse granulocyte–macrophage colony stimulating factor (mGM-CSF) to enrich bone marrow-derived dendritic cells (DCs). Floating cells were removed, and the old media was replaced with fresh complete media containing mGM-CSF every 2 days. Mouse macrophage colony-stimulating factor (mM-CSF) was added to the culture media to generate bone marrow-derived macrophages. The old media was replaced after 3 and 4 days. Immature DCs and macrophages were collected and seeded at a density of 5 × 10⁵ cells/mL in 6-well plates. The cells were incubated with LJ for 48 h. The supernatants were stored at −20 °C until further use for ELISA. The experiments were performed independently in duplicate (3 samples/group), and a representative trial is presented.

### Mixed lymphocyte reaction

An in vitro allogeneic mixed lymphocyte reaction (MLR) assay was conducted to determine the ability of the APCs to induce T cell activation. Allogenic naïve lymphocytes were harvested from the spleen cells of C57bl/6 c mice. Briefly, spleens were mechanically mashed and filtered using a 100-µm cell strainer. After centrifugation at 1600rpm for 5 min and red blood cell lysis, pellets were resuspended in 10% complete media with 1 mM sodium pyruvate, 1× non-essential amino acids, and 55 μM 2-mercaptoethanol. Lymphocytes, prepared DCs, and macrophages were seeded at a density of 2 × 10⁵ cells/well and 1 × 10^4^ cells/well in U-bottom 96-well plates in 200 μL of culture media, so that the ratio of DCs or macrophages to lymphocytes was 1:20. After 5 days of co-culture, the supernatants were stored at −20 °C until used for ELISA. The experiments were performed independently in duplicate (3 samples/group), and a representative trial is presented.

### In vivo immunization and sample preparation

To investigate the effect of LJ in vivo, BALB/c mice were orally administrated 100 µL of phosphates buffered saline (PBS) with or without 10^8^ CFU of LJ (n = 3each). After 12 h, peritoneal and Peyer’s patch cells were collected. Peritoneal cells were collected by washing the peritoneal cavity with 2 mL PBS. Six nodules of Peyer’s patches per mouse were collected, then mechanically mashed, and filtered using a 100-µm cell strainer. After centrifugation at 1600rpm for 5 min, pellets were resuspended in PBS containing 2% FBS for flow cytometry. The experiments were performed independently in duplicate (n=3/group), and the combined data are presented.

### Inflammatory cytokine enzyme-linked immunosorbent assay (ELISA)

Tumor necrosis factor (TNF)-α, interleukin (IL)-6, and IL-12p40 levels were measured using a mouse uncoated ELISA Kit (Invitrogen), and interferon (IFN)- γ and IL-4 levels were measured using the DuoSet ELISA kit (R&D system) according to the manufacturer’s protocols.

### Flow cytometry

**Fig 1:**
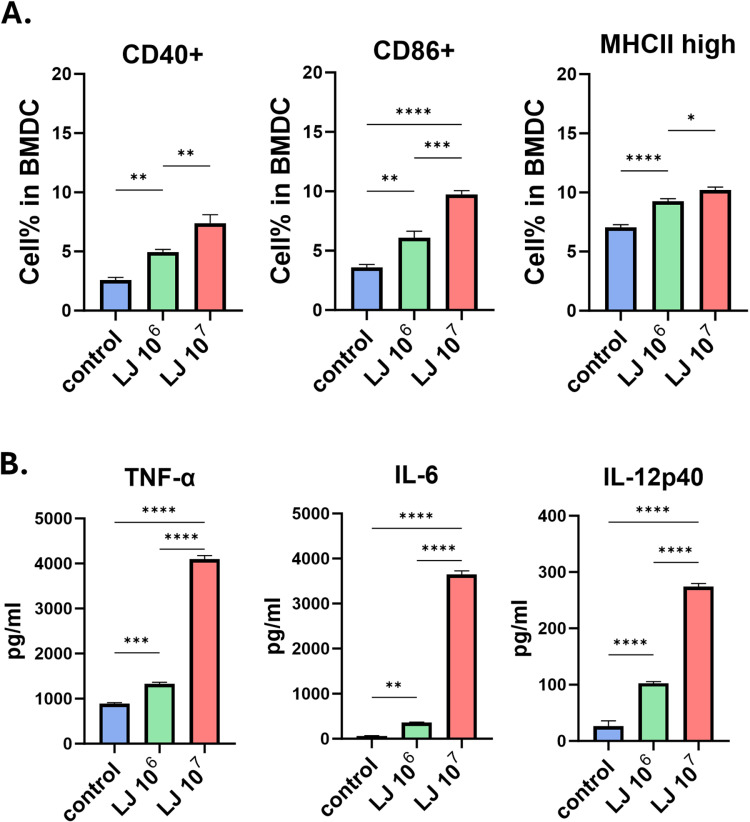
Activation marker expressions and inflammatory cytokine productions of DCs after treated with LJ. DCs were treated with LJ for 48h. Activation markers were determined using flow cytometry (A) and cytokine levels in the supernatant were measured using ELISA (B). All data were shown in mean ± SEM. For statistical analysis, one-way ANOVA was performed. *p < 0.05; **p < 0.01; ***p < 0.001; ****p < 0.0001 between the indicated groups. The experiments were performed independently in duplicate (3 samples/group), and a representative trial is presented.

**Fig 2:**
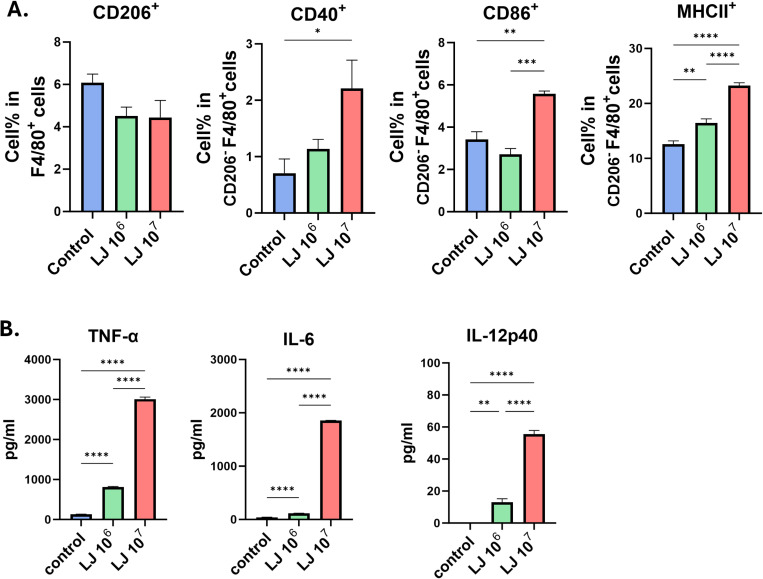
Activation marker expressions and inflammatory cytokine productions of macrophages after being treated with LJ. Macrophages were treated with LJ for 48h. Activation markers were determined using flow cytometry (A) and cytokine levels in the supernatant were measured using ELISA (B). All data were shown in mean ± SEM. For statistical analysis, one-way ANOVA was performed. *p < 0.05; **p < 0.01; ***p < 0.001; ****p < 0.0001 between the indicated groups. The experiments were performed independently in duplicate (3 samples/group), and a representative trial is presented.

For cell phenotype staining, the harvested cells were blocked with anti-CD16/32 (clone 2.4G2) antibody after washing with 2% FBS containing PBS (FACS buffer). Each antibody cocktail was then added to the cells and incubated for 30 min at room temperature in the dark. Intracellular cytokine staining for IFN-γ and IL-4 was performed using the BD Cytofix/Cytoperm kit. The data were acquired using a BD LSR Fortessa and BD FACS DIVA program at the Bio-Health Materials Core Facility, Jeju National University and analyzed using FlowJo software. Cell phenotypes were gated as follow. In vitro DCs: CD11c^+^ with co-stimulatory molecules (CD40 and CD86) MHCII; In vitro M1 macrophage: CD206^-^F4/80^+^ with co-stimulatory molecules and MHCII; In vitro M2 macrophage CD206^+^F4/80^+^; peritoneal cavity and Peyer’s patch DCs: CD45+CD11C+F480- MHCII+; peritoneal cavity and Peyer’s patch Macrophages: CD45^+^CD11b^+^F480^+^Ly6c^-^; Large peritoneal macrophages (LPM): CD45^+^CD11b^+^F480^hi^Ly6c^-^MHCII^-^; Small peritoneal macrophages (SPM): CD45^+^ CD11b^+^F480^int^Ly6c^-^MHCII^+^; Peyer’s patch CD4 T cell: CD45^+^CD3^+^CD4^+^CD8^-^; Peyer’s patch CD 8 T cell: CD45^+^CD3^+^CD4^-^CD8^+^. Table 1 lists the antibodies used in the study. Flow cytometry analysis from Figs 1, 2, 4, and 5 is shown in S1 Fig.

**Table 1:** List of antibodies used in the study.

	Antibody for Flow cytometry
**DC activation marker**	anti-mouse CD11c (clone N418) CD40 (clone 3/23) CD86 (clone GL1) MHC class II (clone I-A/I-E) Live/dead aqua (L/D)
**Macrophage activation marker**	anti-mouse F4/80 (clone BM8) CD40 (clone 3/23) CD86 (clone GL1) MHC class II (clone I-A/I-E) CD206 (clone C068C2) Live/dead aqua (L/D)
**Inflammatory cell in peritoneal cavity**	anti-mouse CD45 (clone 30-F11) CD11b (clone M1/70) CD11c (clone N418) F4/80 (clone BM8) Ly6c (clone AL-21) MHC class II (clone I-A/I-E) Live/dead aqua (L/D)
**T cell population in Peyer’s patch**	anti-mouse CD45 (clone 30-F11) CD3 (clone 17A2) CD4 (clone RM4.5) CD8a (clone 53–6.7) IL-4 (Clone 11B11) IFN- γ (Clone XMG1.2) Live/dead aqua (L/D)

**Fig 3:**
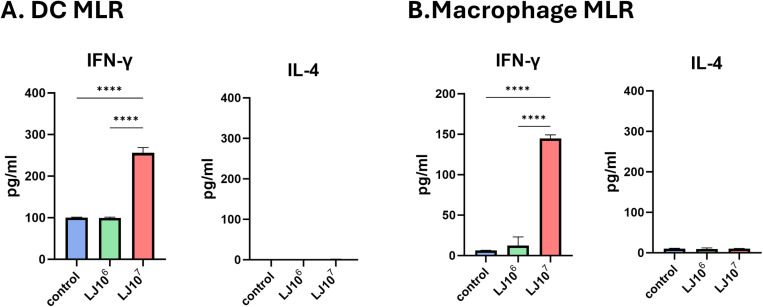
Production of inflammatory cytokines following allogeneic mixed lymphocyte reactions using DCs or macrophages pre-treated with LJ. DCs or macrophages were pre-treated with LJ for 48h. After co-culturing LJ pre-treated DCs and macrophages with allogenic naïve lymphocytes for 5 days, inflammatory cytokine levels in the supernatant were measured using ELISA. Inflammatory cytokine levels after DC MLR (A) and macrophage MLR (B). All data were shown in mean ± SEM. For statistical analysis, one-way ANOVA was performed. ****p < 0.0001 between the indicated groups. The experiments were performed independently in duplicate (4 samples/group), and a representative trial is presented.

**Fig 4:**
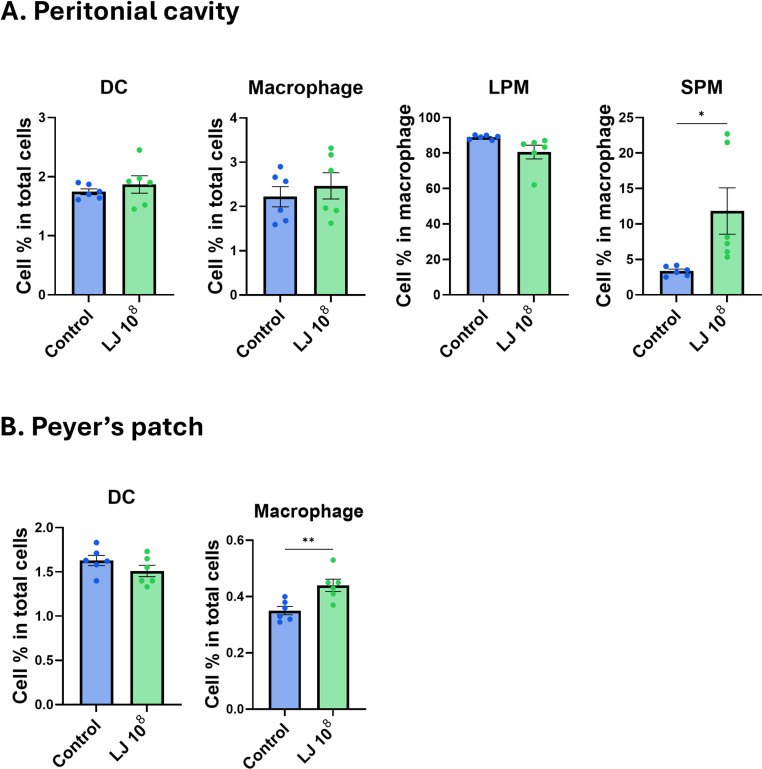
Inflammatory cell population in peritoneal cavity and Peyer’s patch after oral administration of LJ. Cells were collected from peritoneal fluid and Peyer’s patch at 12h after oral administration of 10^8^ CFU of LJ (n=6/group). Populations of DCs and macrophages were determined using flow cytometry. Inflammatory cell populations in peritoneal cavity (A) and Peyer’s patch (B). All data were shown in mean ± SEM. For statistical analysis, unpaired t test was performed. *p < 0.05; **p < 0.01 between the indicated groups. The experiments were performed independently in duplicate (n=3/group), and the combined data are presented.

**Fig 5:**
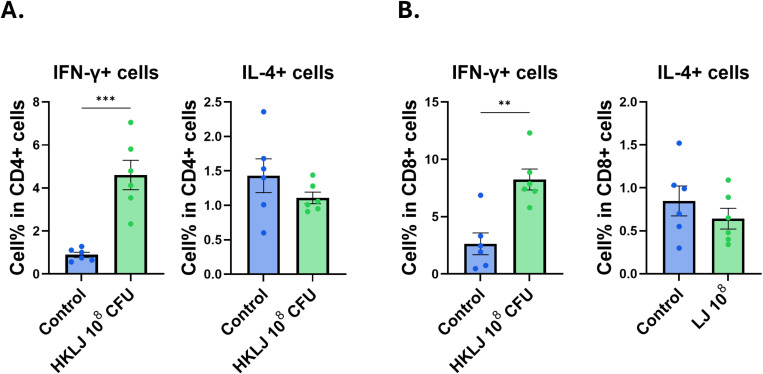
Population of IFN-γ ^+^ and IL-4 ^+^ T cell subsets in Peyer’s patch after oral administration of LJ. Cells were collected from Peyer’s patch at 12h after oral administration of 10^8^ CFU of LJ (n=6/group). IFN-γ ^+^ and IL-4 ^+^ T cell populations in CD4 (A) and CD8 (B) T cells were determined using flow cytometry. All data were shown in mean ± SEM. For statistical analysis, one-way ANOVA was performed. ***p < 0.001 between the indicated groups. The experiments were performed independently in duplicate (n=3/group), and the combined data are presented.

### Statistical analysis

All results are presented as the mean ± standard error of the mean (SEM), and statistical significance was determined using one-way analysis of variance (ANOVA) followed by Tukey’s multiple comparison test. All data were analyzed using GraphPad Prism software 10.09 (GraphPad Software Inc., San Diego, CA, USA).

## Results

### LJ enhanced the maturation of BMDCs and inflammatory cytokine production

To evaluate the effects of LJ on BMDCs, the cells were incubated with 10^6^ and 10^7^ CFU/mL of LJ for 48 h. The dosages of LJ to be used were determined in preliminary experiments (data not shown). The expression of activation markers on the DCs was measured using flow cytometry. LJ significantly increased the expression of MHC II, CD86, and CD40 in DCs dose-dependently (Fig 1A). The production of inflammatory cytokines (TNF-α, IL-6, and IL-12p40) in DCs was also increased by LJ (Fig 1B). These data show that LJ activates DC and enhances the production of inflammatory cytokines.

### LJ induced the polarization of M1 macrophages in BMDM

To evaluate the effects of LJ on BMDMs, cells were incubated with 10^6^ or 10^7^ CFU/mL of LJ for 48 h. The expression of activation markers in macrophages was measured using flow cytometry. LJ significantly increased the expression of MHC II, CD86, and CD40 in CD206^-^ macrophages at a dose of 10^7^ CFU/mL; no significant difference was found in the percentage of CD206^+^ macrophages between the groups (Fig 2A). LJ dose-dependently increased the production of inflammatory cytokines (TNF-α, IL-6, and IL-12p40) in macrophages (Fig 2B). These data show that LJ induces the polarization of M1 macrophages and their functional activation in BMDM.

### BMDCs and BMDMs pre-treated with LJ induced IFN-γ cytokine production in T cells

We co-cultured BMDCs or BMDMs, which were pre-treated with LJ, with allogenic lymphocytes for 5 days to determine their capacity for T cell activation. DCs and macrophages pretreated with 10^7^ CFU/mL of LJ showed significantly higher levels of IFN-γ than control cells (Fig 3A and B). The level of IL-4 was not increased by LJ pretreatment in DCs or macrophages (Fig 3A and B). These data suggest that DCs and macrophages pre-treated with LJ activates T cells in a Th-1 skewed response.

### Oral administration of LJ recruited macrophages in the peritoneal cavity and Peyer’s patch and increased Th-1 T cell population in Peyer’s patch

To determine the effect of LJ in vivo, 10^8^ CFU of LJ was orally administered to the mice. LJ dose was determined by referencing the guidelines. After 12 h, peritoneal and Peyer’s patch cells were collected, and cell populations were analyzed using flow cytometry. The percentage of the small peritoneal macrophage (SPM) population was significantly increased, and the percentage of the large peritoneal macrophage (LPM) population decreased in the LJ-supplemented group (Fig 4A). LPM is a non-inflammatory peritoneal macrophage subset that comprises up to 90% of peritoneal macrophages in the normal state, whereas SPM is a subset of inflammatory peritoneal macrophages, which is increased in inflammatory conditions [23]. There was no difference in the frequency of DCs in the peritoneal cavity (Fig 4A). The population of macrophages in the Peyer’s patch also increased after 12 h of oral administration of LJ, but LJ did not affect the population of DCs (Fig 4B). The percentage of IFN-γ ^+^ cell population significantly increased, whereas the IL-4^+^ cell population decreased in CD4 T cells of Peyer’s patch (Fig 5A and B). These data suggest that the oral administration of LJ mainly recruits macrophages in the peritoneal cavity and activates T cells in Peyer’s patch in a Th-1 skewed response.

## Discussion

Our study showed that LJ shows an immunostimulatory function in DCs and macrophages, which results in the expansion of the IFN-γ cytokine-producing CD4 and CD8 T cell populations. Consistent with the in vitro results, oral administration of LJ resulted in the expansion of IFN-γ cytokine-producing T cells in Peyer’s patches in the small intestine within 12 h. Macrophages are known to be important modulators of T cell immunity in the intestine [24,25] and our data also showed that T cell modulation in Peyer’s patch after LJ oral administration was mainly mediated by macrophages.

Activated macrophages are conventionally categorized into two classifications: M1-like and M2-like. The M1 phenotype predominantly facilitates pro-inflammatory responses. M1 macrophages produce high levels of pro-inflammatory cytokines and Th-1 responses, which are efficacious for protection against pathogens or vaccine response. In contrast, the M2 phenotype is principally associated with anti-inflammatory processes. M2 macrophages are involved in tissue remodeling and immune regulation, which are efficacious for therapeutic applications [26–28]. Thus, the capacity of our LJ to stimulate M1 macrophages and Th-1 responses may prove advantageous when utilized to enhance antiviral immunity or as immune stimulators, such as vaccine adjuvants. However, what makes a difference in the mechanism of LJ JERA01 in activating M1 macrophages from other studies that induce anti-inflammatory effects mediated by M2 macrophage polarization [19] is still unknown. Several factors could account for the observed differences. First, host strain or species variation may influence the effects of LJ. However, this is unlikely to be the primary factor in our case, as we used BALB/c mice, and previous studies using similar mouse models have reported conflicting results [19,29]. Second, the method of LJ preparation—whether live, heat-killed, or lysed—can significantly alter its immunomodulatory effects. For example, metabolites such as short-chain fatty acids, like butyrate, can inhibit histone deacetylases, promoting anti-inflammatory gene expression and M2 polarization [30,31]. Since we used heat-killed LJ without metabolites, we investigated whether bacterial viability contributed to the observed differences. However, our results demonstrated that live LJ JERA01 also induced M1 macrophage polarization in BMDMs and increased inflammatory cytokine production (sup. 2A, B). Thus, we concluded that bacterial viability does not account for the observed effects. Furthermore, a separate in vitro study reported that LJ treatment activated CD206⁺ macrophages in BMDMs, suggesting that the pre-existing immune condition (inflammatory or non-inflammatory) does not significantly alter LJ’s properties [19]. Based on these findings, we assume that strain diversity plays a more critical role in the differences observed.

Lactobacillus species exhibit substantial strain diversity, which can influence their immunomodulatory properties. Differences in surface structures, such as lipoteichoic acids, peptidoglycans, and capsular polysaccharides, may contribute to varying interactions with pattern recognition receptors (PRRs), including Toll-like receptor 2 (TLR2) and nucleotide-binding oligomerization domain (NOD)-like receptors (NLRs) on immune cells. These interactions can elicit distinct immune responses depending on the specific strain and context. For instance, some strains activate the NF-κB pathway via TLR2 and stimulate NLR family pyrin domain containing 3 (NLRP3) inflammasomes, leading to pro-inflammatory cytokine production and Th1 responses [32,33]. In contrast, activation of TLR1/2 signaling can drive M2 macrophage differentiation through STAT3 activation, promoting IL-10 production, inhibiting the TLR4 pathway, and fostering an anti-inflammatory response [19,34].

Additionally, our study has the limitation of not assessing the effects of varying LJ doses, which could influence the observed immune responses. Further research is needed to elucidate the molecular mechanisms underlying LJ JERA01 interactions under different conditions and doses, which would provide a more comprehensive understanding of its immunomodulatory properties.

This research contributes to the understanding of the impact of LJ on intestinal immunity and offers insights into its potential usefulness.

## Supporting information

S1 Fig Flow cytometry analysis from Figs 1, 2, 4 and 5. (PPTX)

S2 Fig Effects of Live LJ on immune cells. Macrophages were treated with live LJ JERA01 for 48h. Activation markers were determined using flow cytometry (A) and cytokine levels in the supernatant were measured using ELISA (B). Inflammatory cytokine levels after macrophage MLR (C). After co-culturing live LJ pre-treated macrophages with allogenic naïve lymphocytes for 5 days, inflammatory cytokine levels in the supernatant were measured using ELISA. All data were shown in mean ± SEM. For statistical analysis, one-way ANOVA was performed. *p < 0.05; **p < 0.01; ***p < 0.001; ****p < 0.0001 between the indicated groups.
(PPTX)
