# Supplementary material for: Lactobacillus johnsonii JERA01 activates macrophages and increases Th-1 T cell population in mouse small intestine
Source: PLoS One. 2025 Apr 24;20(4):e0320946. doi: 10.1371/journal.pone.0320946 (PMC12021164; doi:10.1371/journal.pone.0320946)
Supplement: S1 Fig — (PPTX) [file pone.0320946.s001.pptx]

## Slide 1
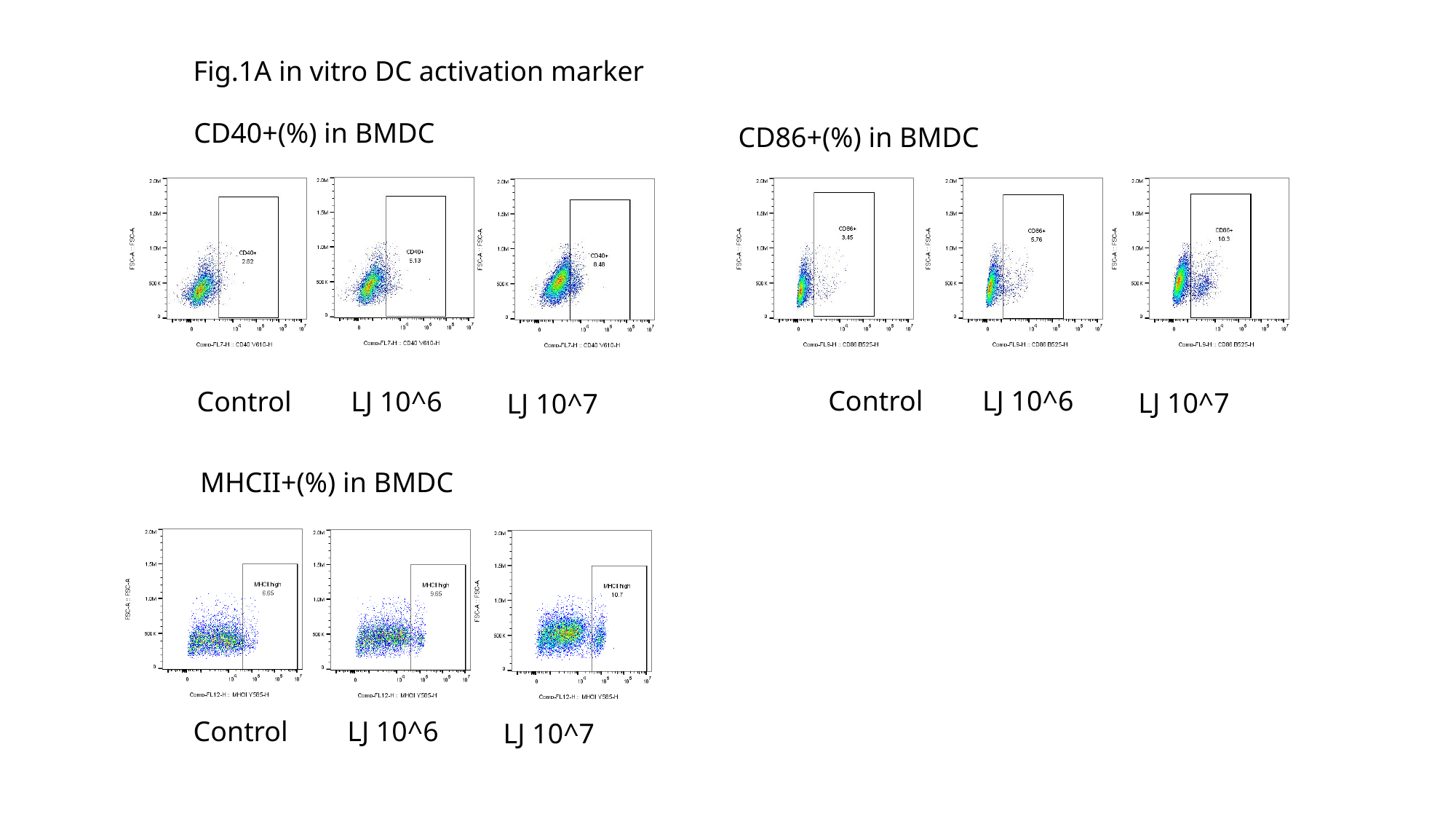

Fig.1A in vitro DC activation marker
CD40+(%) in BMDC
CD86+(%) in BMDC
Control
LJ 10^6
LJ 10^7
Control
LJ 10^6
LJ 10^7
MHCII+(%) in BMDC
Control
LJ 10^6
LJ 10^7

## Slide 2
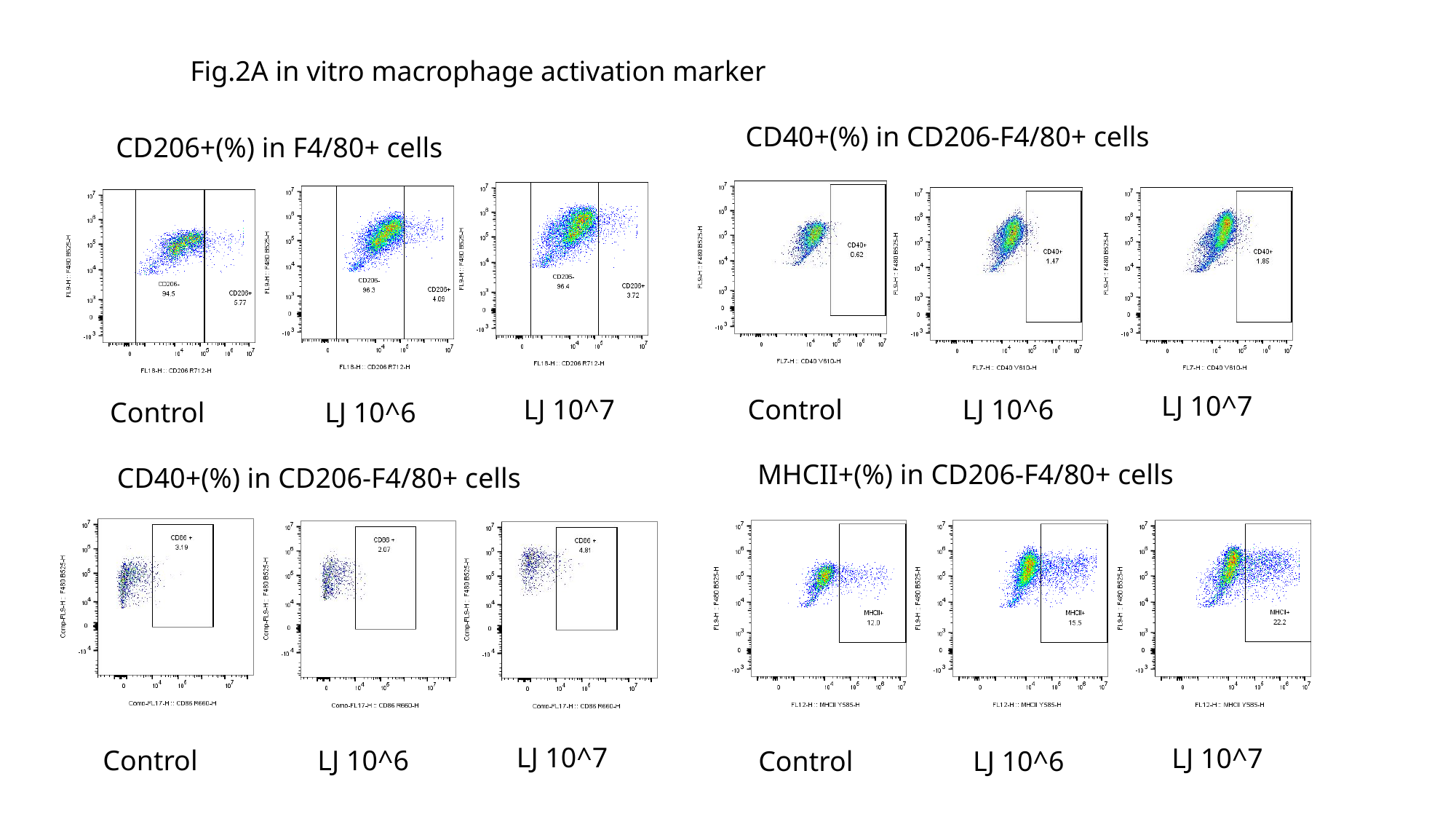

Fig.2A in vitro macrophage activation marker
CD40+(%) in CD206-F4/80+ cells
CD206+(%) in F4/80+ cells
LJ 10^7
Control
LJ 10^6
LJ 10^7
Control
LJ 10^6
MHCII+(%) in CD206-F4/80+ cells
CD40+(%) in CD206-F4/80+ cells
LJ 10^7
Control
LJ 10^6
LJ 10^7
Control
LJ 10^6

## Slide 3
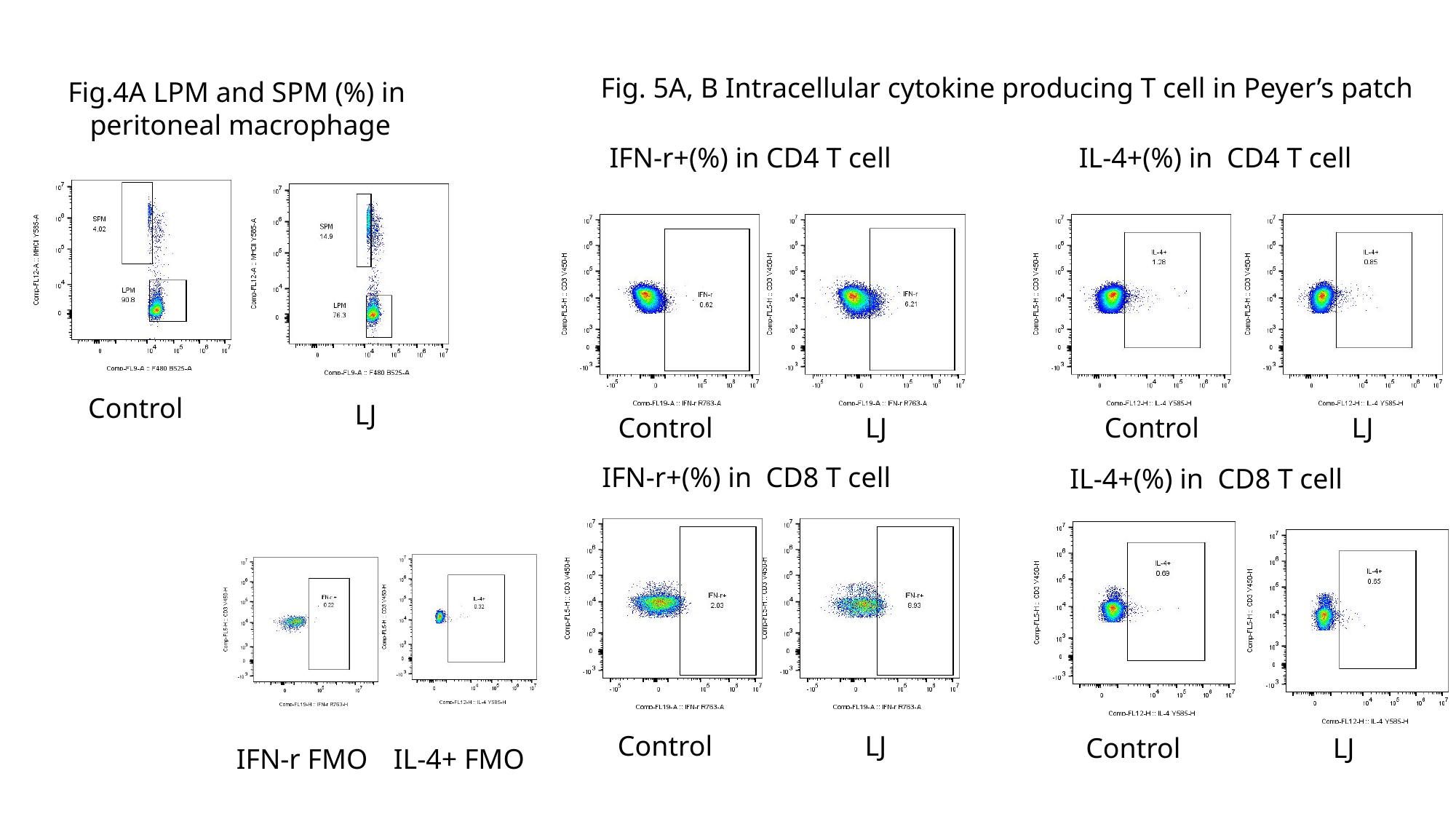

Fig. 5A, B Intracellular cytokine producing T cell in Peyer’s patch
Fig.4A LPM and SPM (%) in
peritoneal macrophage
IFN-r+(%) in CD4 T cell
IL-4+(%) in CD4 T cell
Control
LJ
Control
LJ
Control
LJ
IFN-r+(%) in CD8 T cell
IL-4+(%) in CD8 T cell
Control
LJ
Control
LJ
IFN-r FMO
IL-4+ FMO
