# Supplementary figures and images for: Lactobacillus johnsonii JERA01 activates macrophages and increases Th-1 T cell population in mouse small intestine
Source: PLoS One. 2025 Apr 24;20(4):e0320946. doi: 10.1371/journal.pone.0320946 (PMC12021164; doi:10.1371/journal.pone.0320946)

## Slide 1
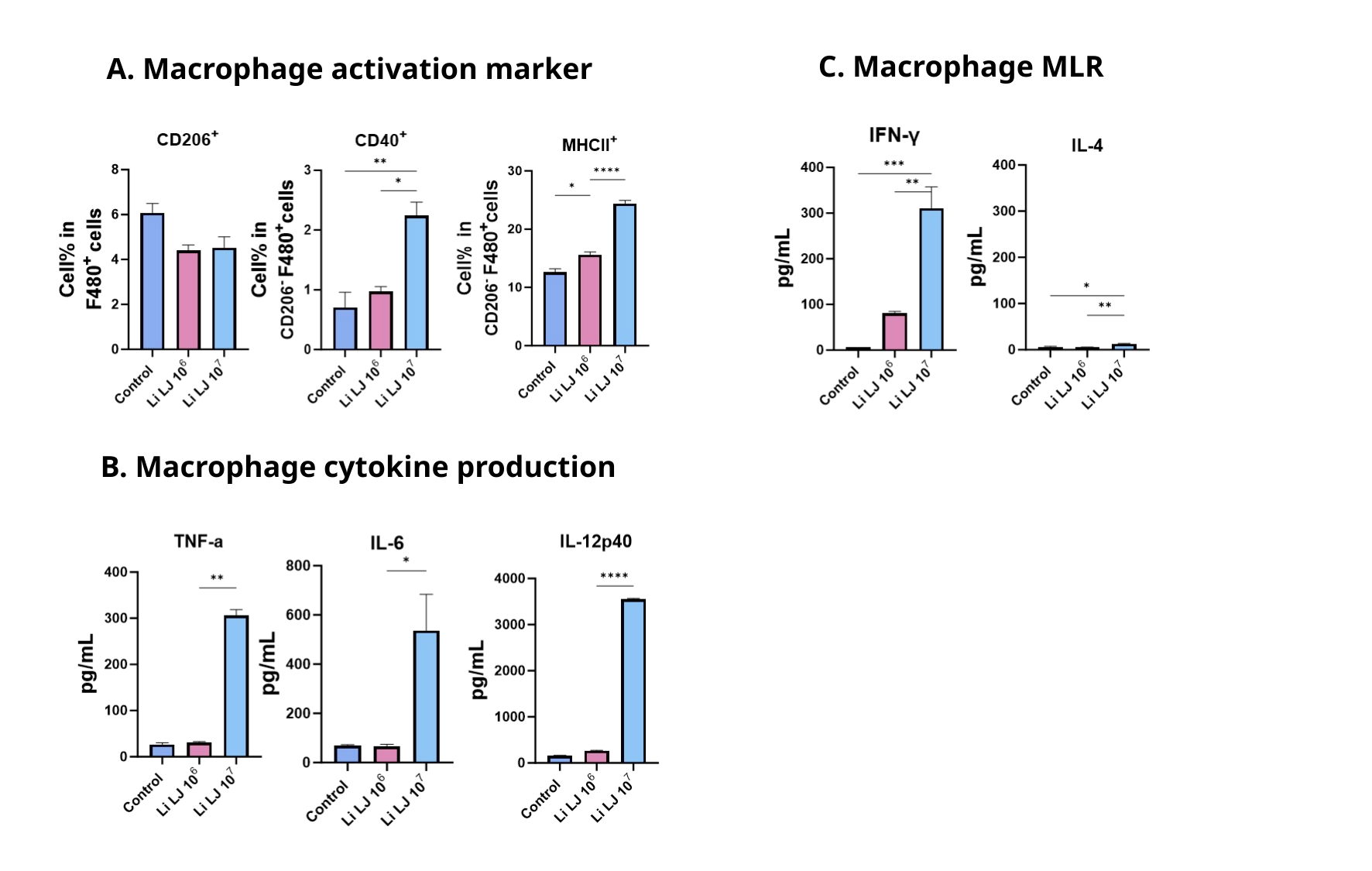

C. Macrophage MLR
A. Macrophage activation marker
B. Macrophage cytokine production

Supplement: S2 Fig — Macrophages were treated with live LJ JERA01 for 48h. Activation markers were determined using flow cytometry (A) and cytokine levels in the supernatant were measured using ELISA (B). Inflammatory cytokine levels after macrophage MLR (C). After co-culturing live LJ pre-treated macrophages with allogenic naïve lymphocytes for 5 days, inflammatory cytokine levels in the supernatant were measured using ELISA. All data were shown in mean ± SEM. For statistical analysis, one-way ANOVA was performed. *p < 0.05; **p < 0.01; ***p < 0.001; ****p < 0.0001 between the indicated groups. (PPTX) [file pone.0320946.s002.pptx]
